# What You Know, What You Do, and How You Feel: Cultural Competence, Cultural Consonance, and Psychological Distress

**DOI:** 10.3389/fpsyg.2017.02355

**Published:** 2018-01-15

**Authors:** William W. Dressler, Mauro C. Balieiro, José E. dos Santos

**Affiliations:** ^1^Department of Anthropology, The University of Alabama, Tuscaloosa, AL, United States; ^2^Department of Psychology, Paulista University, Ribeirão Preto, Brazil; ^3^Department of Internal Medicine, Faculty of Medicine, University of São Paulo, Ribeirão Preto, Brazil

**Keywords:** cultural consensus analysis, cultural competence, cultural consonance, psychological distress, Brazil

## Abstract

Describing the link between culture (as a phenomenon pertaining to social aggregates) and the beliefs and behaviors of individuals has eluded satisfactory resolution; however, contemporary cognitive culture theory offers hope. In this theory, culture is conceptualized as cognitive models describing specific domains of life that are shared by members of a social group. It is sharing that gives culture its aggregate properties. There are two aspects to these cultural models at the level of the individual. Persons have their own representations of the world that correspond incompletely to the shared model—this is their ‘cultural competence.’ Persons are also variable in the degree to which they can put cultural models into practice in their own lives—this is their ‘cultural consonance.’ Low cultural consonance is a stressful experience and has been linked to higher psychological distress. The relationship of cultural competence *per se* and psychological distress is less clear. In the research reported here, cultural competence and cultural consonance are measured on the same sample and their associations with psychological distress are examined using multiple regression analysis. Results indicate that, with respect to psychological distress, while it is good to know the cultural model, it is better to put it into practice.

## Introduction

One of the enduring theoretical and methodological problems in the social sciences has been to describe how culture and the individual are linked (Chentsova-Dutton et al., 2010; Markus and Kitayama, 2010; Dressler, 2018). There are a variety of questions that demand an explication of this link, with health perhaps the most obvious (Kagawa Singer et al., 2016). How is variation in individual well-being related to culture?

Dressler (2007, 2018) developed a theory of ‘cultural consonance’ to address this question. Cultural consonance is the degree to which individuals, in their own beliefs and behaviors, approximate the prototypes for belief and behavior encoded in cultural models. Low cultural consonance is associated with greater psychological distress (Dressler et al., 2017) and other health outcomes (Dressler, 2018).

The measurement of cultural consonance is derived from cultural consensus analysis (Romney et al., 1986), a technique for determining both the degree to which knowledge in a specific cultural domain is shared and the degree to which an individual’s knowledge of the domain is linked to the group or aggregate-level shared knowledge, referred to as that individual’s ‘cultural competence.’ In our prior work we have verified cultural consensus in one sample, then measured cultural consonance and health outcomes on an independent sample. This begs the question, however, of how cultural competence (individual agreement with the cultural consensus) and cultural consonance (individual enactment of consensus knowledge in the domain) may overlap in relation to health outcomes such as psychological distress. This question will be examined in this paper employing data recently collected in urban Brazil.

## A Cognitive Theory of Culture

The general idea that the match (or, often, a mis-match) between an individual’s beliefs, values, and behaviors, and the normative expectations for belief, value, and behavior in a given sociocultural setting might be related to health, is not a new one. Theories proposed to describe this link include ‘social behavior’ (Sapir, 1927); ‘cultural congruity’ (Cassel et al., 1960); ‘person-environment fit’ (Caplan and Harrison, 1993); the ‘cultural norm hypothesis’ (Chentsova-Dutton et al., 2010); ‘cultural fit’ (De Leersnyder et al., 2015); ‘intersubjective culture’ (Wan et al., 2007); and, ‘cultural consonance’ (Dressler, 2018). These approaches are consistent in the sense that all view individuals as living within an environment of shared meaning and understanding—in short, ‘culture’—while the correspondence of the individual to that culture, in terms of his or her own beliefs and behaviors, is imperfect. Where the approaches differ is mainly in how these various constructs are operationalized. In the measurement of cultural consonance, a radically ‘emic’ approach is adopted (see below).

The concept and measurement of cultural consonance is derived from a cognitive theory of culture (D’Andrade, 1995; Kronenfeld, 2011). This approach is based on Goodenough’s (1956) definition of culture as that which one must know in order to function effectively in a given social setting. Shared knowledge enables us to interpret the behavior of others and to initiate behavior that is intelligible to others in social interaction. This knowledge is used to culturally construct the conditions of life that seem to have an existence independent of the people making up a society, yet ultimately these seemingly external conditions are based on the collective understanding that this is indeed the way the world works (Searle, 1995).

Knowledge is stored cognitively in the form of mental representations of specific cultural domains. A cultural domain is an organized sphere of discourse (Weller and Romney, 1988); it is, in short, something about which people talk. The representation of a cultural domain is a mental model that contains the elements of the domain along with semantic, functional, and causal associations among those elements. It is a stripped-down, skeletal representation that includes one or more prototypes or best exemplars of the domain. What makes these mental models *cultural* is that they are shared among and learned by individuals. A given individual’s model of some domain will be composed of two parts: (a) the cultural component, learned and variably shared with others; and (b) a personal component, derived from an individual’s unique biography. For example, American students tend to share a cultural model of food, based on features describing how meals are constructed and the healthfulness of foods. At the same time, due to regional and family traditions, there are unique components to any one individual’s model (Dressler, 2018, p. 67–76).

The utility of a theory of cultural models was enhanced considerably by the introduction of cultural consensus analysis (CCA) by Romney et al. (1986; Batchelder and Romney, 1988; Weller, 2007). Using CCA, shared knowledge in a particular cultural domain can be empirically verified. Once the elements of a cultural domain have been identified, along with the features that individuals use to organize those elements, respondents can be asked to rate or rank those elements in terms of those features. For example, in our work in Brazil we elicited the characteristics of a family, and these in turn were rated in terms of their importance for having a good family (Dressler et al., 2005b). The aim of CCA is to determine if the responses to the rating task are sufficiently similar from person to person to justify the inference that the respondents are drawing on a common pool of knowledge (or, culture). CCA works from a correlation matrix of respondents to estimate a linear function summarizing the similarities in their ratings. If most of the members of the sample are highly correlated with this linear function, the inference regarding a common culture is justified. These correlations of individuals with the linear function assess their cultural competence, or the degree to which their understanding of the domain corresponds to the aggregated knowledge. The responses of the individuals can then be combined in a weighted average (weighted by each respondent’s competence) to determine how a reasonably knowledgeable, or culturally competent, member of that society would answer those questions. This weighted average of responses is referred to as the ‘cultural answer key’ in consensus analysis (Romney et al., 1986).

Cultural consensus analysis makes use of factor analytic methods to estimate cultural competence, although it is not a traditional factor analysis. Rather, CCA uses the minimum residual method to estimate the diagonal of the correlation matrix of respondents, which is in turn used to estimate cultural competence. The ratio of the first-to-second eigenvalue of the correlation matrix is also useful as a summary measure of the degree of shared knowledge. Traditionally, a ratio of 3:1 or larger is considered to be indicative of a shared cultural model (Romney et al., 1986; Batchelder and Romney, 1988). The cultural consensus model has been widely used and proven to be robust (Batchelder and Anders, 2012). Furthermore, CCA can be used with relatively small samples, especially when agreement regarding a cultural domain is very high (Romney et al., 1986). This is true in part because the focus for CCA is on relatively homogeneous cultural domains, in the sense that the items making up the domain are closely associated in defining that domain.

Cultural consensus analysis provides a systematic and operationally explicit way of verifying the sharing of knowledge, along with estimates of how strongly it is shared and how it is socially distributed (Gatewood, 2012). Cultural models that are widely shared can be described, and with recent innovations such as the analysis of residual agreement (Dressler et al., 2015), cultural models that are weakly shared, highly contested, or have multiple emphases can be systematically explored. Furthermore, CCA accounts for the dual nature of culture in a non-mysterious way. Culture is a term that applies to social aggregates (e.g., societies, organizations, professions), yet, at the same time, the fundamental referent of the term must be the individual. Individuals do indeed carry a version of a cultural model around in their heads, but the aggregate or collective model is not a mere averaging of what individuals know. It is rather a weighted composite that takes into account the social distribution of knowledge; hence, the term culture literally refers to aggregates in a sense in which it does not refer to individuals.

People, however, do not simply know or understand things; they *do* things as well. How is culture reproduced in what people actually do in their day-to-day lives? The concept of cultural consonance, as individuals’ approximation to the cultural model in their own belief and behavior, measures cultural fit. Measures of individual belief and behavior can be constructed using the cultural answer key and directly assess how closely individuals match the cultural model.

## Cultural Consonance and Health

Over the past 25 years, cultural consonance has been examined in relation to health outcomes in a variety of settings. The hypothesis guiding this work is straightforward. There is a set of cultural models that encode major life goals in specific social settings. Together, these represent normative expectations about how life is to be lived in those settings, especially when these cultural models are strongly shared. The person with low cultural consonance, however, is not achieving those goals. He or she is seen to be, and sees him- or herself as, socioculturally marginal. This is likely to be a stressful condition and to be related to worse health outcomes.

The original work on cultural consonance was carried out in urban Brazil by Dressler et al. (1997, 1998) examining blood pressure. The cultural domains of interest were lifestyle (material goods and leisure time activities contributing to having a good life) and social support. These cultural domains capture in experience-near terms the broader theoretical domains of social class and social integration. Higher cultural consonance in lifestyle and higher cultural consonance in social support were associated with lower blood pressure, controlling for standard covariates (age, sex, body mass index, socioeconomic status) and for dietary intake (sodium, potassium, saturated fat, and alcohol). These associations were then replicated in a second study carried out 10 years later (Dressler et al., 2005a). Also, these associations were replicated independently in the African–American community in the rural Southern United States (Dressler and Bindon, 2000); among African–American adolescents in suburban Chicago (Sweet, 2010); and among the Tsimane’, an ethnic group in low-land Bolivia (Schultz, 2014).

Cultural consonance in multiple cultural domains has been examined in relation to psychological distress. In urban Brazil, in addition to lifestyle and social support, these domains included family life, national identity, occupational and educational aspirations, and food (Dressler et al., 2007a). Dressler et al. (2017) recently showed that these specific cultural domains are integrated via a broader cultural construct of ‘goals in life,’ and a summary measure of cultural consonance across all the domains was associated with lower depressive symptoms. Other studies have replicated the association of cultural consonance and health in a variety of settings (Snodgrass et al., 2011; Dengah, 2014). Finally, in longitudinal studies Dressler et al. (2007b) and Reyes-García et al. (2010) found change over time in cultural consonance to be associated with change in depressive symptoms and mood states, respectively.

In general, the cultural consonance hypothesis appears to be robust.

## Cultural Competence, Cultural Consonance, and Health

The measurement of cultural consonance is a two-stage process in research. A cognitive theory of culture takes what is termed an ‘emic’ approach in anthropology (Pike, 1954). That is, the point of research is to discover what is salient and meaningful to members of a social group and to employ those constructs in analysis, rather than employing measures that are salient and meaningful primarily to researchers. A cultural model therefore must be elicited and the features structuring the domain discovered before a measurement can be developed. An emic analysis starts with the assumption that the investigators do not themselves know, for example, what represents a valued lifestyle or a good family in the research setting; these elements of the cultural model must be discovered and elicited from the respondents themselves. A measurement of cultural consonance derived from this cultural domain analysis thus orders persons along a continuum defined in the terms that they themselves use to talk about that domain. For this reason, we have referred to measures of cultural consonance as having high ‘emic validity.’ Emic validity is demonstrated through the derivation of the measure (Dressler and Oths, 2014).

In the research we have carried out thus far, the cultural domain analyses, including cultural consensus analysis, were conducted with one sample, the results of which were used to develop the measures of cultural consonance. The measures of cultural consonance were then used with a second sample to test the association of cultural consonance and health outcomes. Logically, this means that a respondent’s own cultural competence (i.e., how he or she agrees with the group) will not contaminate his or her self-report of belief or behavior within that domain. Logistically, this reduces the burden on the respondent and the interviewer, especially when cultural consonance is being measured in multiple cultural domains.

At the same time, this strategy eliminates the potential for examining the relationship between cultural competence in a cultural model and cultural consonance in that same analysis—that is, knowing the model vs. enacting the model. It also eliminates the potential for testing the association between cultural competence and a health outcome, or for examining the association of cultural consonance and that health outcome while controlling for cultural competence. There is some evidence that cultural competence itself is associated with health outcomes. Copeland (2017) conducted research among impoverished women in the marginal communities of Nairobi, Kenya who were HIV positive. Typically these women, often from rural areas, had been expelled from their families following discovery of their seropositive status. They had no access to treatment by antiretrovirals through the official health system because they were considered to be too high risk. Through support provided by churches, non-governmental organizations, and each other, women developed their own models for managing their HIV status. These models consisted of a mix of straightforward public health practice, emotional support, and unorthodox practices (e.g., faith healing).

Copeland (2017) found that there was a cultural model of HIV management shared among the women, and that women’s cultural competence in the model increased with the length of time they had been in Nairobi. In a cross-sectional sample, she found that higher cultural competence in the model was associated with lower reported depressive symptoms, lower perceived stress, and fewer reported opportunistic infections, concluding that sheer knowledge of this cultural model was associated with better health status.

The aim of the study we report here is to build on this finding from Copeland’s research. Specifically, using data collected in urban Brazil, we will examine both cultural competence and cultural consonance in five cultural domains in relation to reported psychological distress. This research is self-consciously exploratory. We are interested to see what happens to the association of cultural consonance and psychological distress when cultural competence is controlled for, and to see if higher cultural competence itself is associated with lower reported psychological distress.

## The Research Setting

Our research in Brazil has been carried out over the past 25 years in the city of Ribeirão Preto (pop. = 600,000) in the interior of the state of São Paulo. This community has been described in some detail in other places (e.g., Dressler et al., 2017; Dressler, 2018). It is a fairly prosperous community serving as a center for finance, education, and health care for the northern region of the state of São Paulo. The surrounding countryside is some of the richest agricultural land in Brazil and supports industrial cultivation of citrus, soy, coffee, and sugar cane. Nevertheless, persistent and high social inequality exists here as well, as in the rest of Brazil.

To account for this inequality, our research has employed two strategies. In cultural domain analyses, we have either systematically sampled by completed education, which is a good indicator of social class in Brazil, or we have sampled from neighborhoods differing in socioeconomic status. In survey work we have drawn stratified random samples of households and individuals using neighborhoods as economic strata. Three major studies have been carried out using this approach, in 1991 (Dressler et al., 1997), 2001 (Dressler et al., 2005a), and 2011 (Dressler et al., 2017). Each study extended over a 2–3 years period of data collection. In 2011 we conducted a follow-up that provides the data for this paper.

The sampling strategy for our survey research involved the selection of four neighborhoods to serve as economic strata. The poorest neighborhood was originally a *favela* or so-called ‘squatter settlement.’ These communities arise when individuals ‘invade’ a piece of unused land, constructing houses out of whatever material can be obtained. As a *favela* develops it takes on more permanence as residents liberate resources from passing water, sewer, and electrical lines. Shortly after our 1991 study this *favela* was razed and the residents moved to a kind of public housing project, consisting of a neighborhood of 2–3 room cinder block houses. Residents are unstably employed as agricultural workers, unskilled construction workers, and domestic servants.

The second neighborhood started in the late 1980s as a *conjunto habitacional* (literally ‘living together’), which is a housing development funded by a public-private initiative. Residents qualify for low-interest mortgages by demonstrating stable employment. The neighborhood started with 4-room cinder block tract houses, but quickly took on a distinctive feel as residents added rooms, garden walls, and sometimes even second stories to their houses. Now the neighborhood has a small commercial district. Residents are employed as technicians, practical nurses, primary school teachers, and skilled factory workers.

The third neighborhood is an old, traditional middle-class neighborhood that dates back to the beginning of the 20th century. Separated from the city proper by a small river and major avenue, the neighborhood was home to immigrants arriving from Italy and Spain, and it retains a distinctive Mediterranean feeling. The community boasts its own Catholic Church on a large square, and it has an extensive commercial district. Neighborhood residents are employed as teachers, nurses, lower-level managers, and small business owners.

The fourth neighborhood is an upper-middle class *condomínio* (‘condominium,’ as gated communities are referred to in Brazil). It sits on the edge of the city, close to a major university campus. Houses are large with extensive gardens, tended by domestic servants who might live in the poorest neighborhood described above. Residents of the *condomínio* are owners of large businesses, lawyers, university professors, and physicians.

While these sampling strata do not exhaust the range of economic inequality in Brazil, they do provide sufficient variation in social class to effectively take such inequality into account.

### Measuring Cultural Competence

The current study builds on our entire 25 years of research in Brazil, and specifically on cultural domain analyses conducted in 2001 and replicated in 2011. These analyses have been described in detail elsewhere, so only an overview will be provided here (Dressler et al., 2005b, 2015; Dressler, 2018). The cultural domains of interest were selected for both theoretical and ethnographic relevance. As Dressler (2018) has argued, a commitment to an emic orientation does not preclude the selection of cultural domains for study on the basis of theory or previous research. It does, however, require that the cultural salience of those domains be demonstrated in the specific social context of the study. The cultural domains of lifestyle, social support, and family life were selected both because of their theoretical relevance to health outcomes and because they are domains that emerge in everyday conversation in Brazil. We also included the cultural domain of national identity, since there is a long history of interest in ‘Brazilian-ness’ in scholarly literature in Brazil. Finally, on the basis of subsequent research (Dressler et al., 2017), we added the domain of occupational and educational pursuits.

The cultural domain analyses proceeded as follows in 2001 [Dressler et al., 2005b; see Borgatti (1999) for a general discussion of these methods]. First, 43 individuals were asked to free list elements of each domain. For example, in the domain of lifestyle we asked respondents to list material goods and leisure time activities that were necessary for ‘having a good life.’ In the domain of national identity we asked them for characteristics that ‘make Brazilians Brazilian.’ Although the number of elements varied from one domain to another, totals in excess of 100 terms or phrases were not unusual. Once synonyms were accounted for, 20 to 40 terms were selected in each domain on the basis of salience, which is calculated from the proportion of persons who list the term and the rank order in which it appears. (More specific detail on samples and items are presented in Supplementary Table 1).

Next, a sample of 40 respondents was tasked with unconstrained pile sorts in each domain. Pile sorts are useful for eliciting the distinctive features or dimensions that individuals use to distinguish among the terms within the domain. Not surprisingly, a general evaluative dimension emerged within each domain. For example, people distinguished among lifestyle elements that were or were not deemed ‘important’ in having a good life. Similarly, respondents referred to characteristics of the family as more or less important for having a family, or various sources of social support as more or less important in providing help and assistance in response to specific kinds of felt need. In the domain of national identity sample members identified different characteristics as more or less authentically Brazilian.

A CCA was carried out in each domain, with respondents (*n* = 66) either rating on a 4-point scale (lifestyle, national identity, occupational and educational aspirations) or ranking (family life, social support) the elements of each domain according to importance. It should be noted how the questions were framed. Respondents were asked to think about importance ‘to people in general, in this community’ when rating or ranking elements. Cultural consensus was verified in each domain. In the domain of national identity and occupational and educational aspirations, the consensus was fairly modest, while in the domains of lifestyle, social support, and family life the consensus was quite strong (see Supplementary Table 1; Dressler et al., 2005b).

A cultural competence coefficient is estimated for each respondent. Like a correlation coefficient or a factor loading, this varies between 0 and 1, and the higher the coefficient, the greater that respondent’s knowledge of the cultural domain in the sense that he or she agrees more strongly with all other respondents concerning the importance of elements in each cultural domain.

In our study in 2011, we replicated the cultural domain analyses to determine if there had been any significant change in the cultural models in the intervening 10 years (the sample size was *n* = 40, no overlap with the 2001 sample). The cultural consensus in each domain, both in terms of the ratio of the first-to-second eigenvalues and in terms of mean cultural competence, was within a few decimal points in 2011 of values observed in 2001, attesting to a remarkable stability, in this context and in these domains, of cultural models.

In 2011 we also carried out a cultural domain analysis of an integrative construct of ‘goals in life’ (Dressler et al., 2017). Each of the domains outlined above emerged in this analysis, along with a domain we had not previously examined: occupational and educational aspirations. This domain was included in subsequent data collection. A small study to confirm cultural consensus in the domain of occupational and educational aspirations was carried out in 2011.

Any cultural domain analysis must be sensitive to potential intracultural diversity. That is, are there subgroups that might differ in their cultural models in these domains? We did not have any clear anticipation of intracultural diversity, but age, gender, and social class differences seemed possible. Therefore, the samples on which cultural consensus was tested were selected to vary along these parameters. There were some differences in residual agreement among these groups, but this did not alter the overall cultural consensus (Dressler et al., 2015).

### Measuring Cultural Consonance

Scales of cultural consonance were developed in 2001 in the domains of lifestyle, social support, family life, and national identity. The scale of cultural consonance in occupational and educational pursuits was developed in 2011. Each was based on the cultural answer key for CCA and each scale varied slightly, depending on the domain. This answer key provides the aggregate rating or ranking of the importance of each element in a specific cultural domain.

To measure cultural consonance in lifestyle, respondents were asked about ownership of material goods and the frequency with which they engaged in particular leisure time activities that had been rated in the CCA. The number of goods they possessed and the activities they regularly engaged in that had received a consensus rating of at least ‘somewhat important’ in the CCA was counted and divided by the total number of items rated as somewhat important. To measure cultural consonance in social support, respondents were asked to rank from most likely to least likely the sources of social support they would seek in response to a set of common problems. Each individual’s ranking was then correlated with the consensus rankings and this correlation was used as the measure of cultural consonance.

For cultural consonance in family life, a set of 18 Likert-response items was developed in which an element of the consensus model of family life served as the subject of a proposition (e.g., “People in my family are hard-workers”), and respondents were asked to agree or disagree with the proposition on a 4-point scale. Then, each item was weighted by the rank of that element in the cultural consensus answer key. This scale has high internal consistency reliability (Cronbach’s alpha = 0.89). Similar scales were developed for occupational and educational pursuits (alpha = 0.81) and for national identity. In the domain of national identity there were subsets of elements that described positive characteristics of the Brazilian people (e.g., they are friendly) and Brazilian institutions (people love *carnaval*). There was also a subset of elements that painted a less than flattering picture of Brazilians (as people who are lazy and ready to take advantage of others). It was the latter elements that formed a consistent scale (alpha = 0.60) describing a ‘cultural cynicism,’ given that it captured a dimension critical of Brazilian national identity. These scales are described in detail elsewhere (Dressler et al., 2005b; Dressler, 2018).

Measures of cultural competence and cultural consonance were then employed in the current study, conducted between 2011 and 2014.

## Materials and Methods

The research protocol was approved by human subjects boards at the University of Alabama and the University of São Paulo - Ribeirão Preto.

### Sampling

The original sample for the 2011 study consisted of 477 respondents randomly selected within the four neighborhoods described above (see Dressler et al., 2017). As a follow-up to that study, in order to examine cultural competence and cultural consonance on the same sample, we randomly sampled 25 respondents from each neighborhood from the 477 respondents originally interviewed. These respondents were then re-interviewed with a new interview schedule that included questions necessary to measure both cultural competence and cultural consonance. We were able to successfully follow up 64 respondents, and these form the sample for this paper.

### The Dependent Variable and Covariates

The main covariates for this study include age (in years); gender (coded 0 = female; 1 = male); and socioeconomic status. The latter was a composite of reported family income, respondent’s years of education, and neighborhood of residence (a set of dummy variables). These all loaded a single principal component, and the principal component score was used to measure socioeconomic status.

The dependent variable, referred to as ‘psychological distress,’ is a composite of depressive symptoms and perceived stress. Depressive symptoms were assessed with the Center for Epidemiologic Studies Depression Scale (CES-D), as translated into Portuguese and validated by da Silveira and Jorge (2000). It has good internal consistency reliability in our research (alpha > 0.90). Perceived stress was assessed with the 10-item version of Cohen’s Perceived Stress Scale (Cohen et al., 1983). Our research group translated and back-translated this scale in 1991 (Dressler et al., 1998) and we have used it multiple times. It has both good internal consistency reliability (alpha > 0.75) and construct validity (correlations with depressive symptoms > 0.65) in various samples. The scales were standardized and summed as a composite index of psychological distress (see Dressler et al., 2007a).

### Measuring Cultural Competence in the Current Study

To measure cultural competence, respondents rated or ranked elements of the cultural domains as described above for the original cultural consensus analyses. Again, they were instructed not to think solely in personal terms, but rather to think about the relative importance of the elements in terms of how people in the community in general might respond. In order to limit contamination of the scales of cultural consonance by the measurement of cultural competence, these were separated in the interview schedule by the collection of sociodemographic and psychological data. Cultural competence coefficients in each domain for each respondent were estimated by combining the current subset of data with the samples on which cultural consensus was previously tested in 2001 and 2011. This gave a total sample size of 176 and estimating the cultural competence coefficients in this way provides more stable estimates.

Cultural competence coefficients calculated for each respondent for each cultural domain vary from 0.00 to 1.00 (with the rare exceptions of a few negative competencies) and assesses how knowledgeable the respondent is in that domain, in the specific sense of the degree to which he or she agrees with others in their evaluations of elements of each domain.

### Measuring Cultural Consonance in the Current Study

Scales of cultural consonance described above were used:

• Cultural consonance in lifestyle: Respondents reported whether or not they owned culturally salient material goods (14 items) and if they engaged in culturally salient leisure activities (7 items). Cultural consonance was calculated by counting the number of items owned by the individual and the number of frequent leisure behaviors reported by the individual. That sum was then divided by the total to yield a proportion. The closer to 1.0, the more the respondent’s own lifestyle matches that of the consensus model.
• Cultural consonance in social support: Respondents ranked seven potential supporters (family, friend, colleague, church member, health professional, a specialist in the area, other person) in the order in which they would seek support from them for each of nine problems (needing a ride, losing your job, relationship problems, problems with your children, problems at your job, needing money, illness, depression, unemployment). Cultural consonance was calculated as a simple correlation between individual rankings and the consensus rankings.
• Cultural consonance in family life: An 18-item, Likert-response scale was created from the results of the cultural domain analysis from 2001. Each item was also weighted by its salience in the consensus analysis. Sample items include the following: (a) “Sometimes I wish my family were more organized” (reverse coded to assess family structure and organization); (b) “In my family we feel close to one another” (to assess emotional climate); and, (c) “When I do something, I don’t think about my family” (to assess threats to the family). The scale has quite high internal consistency reliability (alpha = 0.89).
• Cultural consonance in national identity: An 8-item, Likert-response scale was created. As described elsewhere (Dressler et al., 2005b, 2007b), the survey items with the highest internal consistency were those that described negative social characteristics of Brazilians (e.g., “Many people are just too lazy to get ahead in life”). This scale has acceptable internal consistency reliability (alpha = 0.60) and represents “cultural cynicism.” That is, those individuals who endorse more of the items have a more cynical view of Brazilians and Brazilian life, but it is a distinctly culturally constructed cynicism (see Da Matta, 1985).
• Cultural consonance in occupation and education: This was a 13-item scale in which respondents rated, on a 4-point scale, their agreement or disagreement with statements regarding their satisfaction with occupational and educational opportunities. Sample items include: “I feel satisfied with the professional opportunities I have had;” “In my life, to improve my knowledge is a very important goal;” and, “I feel that I have achieved success in my life.” The scale has acceptable internal consistency reliability (alpha = 0.81).

## Results

### Analysis and Results within Individual Cultural Domains

Descriptive data for all variables used in this analysis are shown in **Table 1** for the sample as a whole, and broken down by the two lower income vs. the two higher income neighborhoods (neighborhoods were combined as two groups due to the relatively small sample size).

**Table 1:** Descriptive statistics (variables marked with asterisks differ significantly between neighborhoods).

	Total sample (*n* = 64)	Lower income neighborhoods (*n* = 31)	Higher income neighborhoods (*n* = 33)
Psychological distress	0.00 (± 1.00)	0.14 (± 0.93)	–0.13 (± 1.05)
Age	54.2 (± 14.3)	53.6 (± 12.3)	54.7 (± 16.0)
Gender (% male)	27.0	22.6	30.3
SES^a,^∗∗∗^^	0.00 (± 1.00)	–0.59 (± 0.89)	0.56 (± 0.74)
Cultural competence-lifestyle	0.57 (± 0.23)	0.62 (± 0.21)	0.51 (± 0.25)
Cultural competence-social support	0.55 (± 0.18)	0.52 (± 0.16)	0.57 (± 0.19)
Cultural competence-family life	0.80 (± 0.11)	0.80 (± 0.11)	0.80 (± 0.11)
Cultural competence-national identity	0.41 (± 0.41)	0.43 (± 0.23)	0.39 (± 0.22)
Cultural competence-occupation/education^∗^	0.53 (± 0.29)	0.46 (± 0.28)	0.64 (± 0.29)
Cultural consonance in lifestyle	83.4 (± 0.21)	79.5 (± 22.5)	87.1 (± 19.4)
Cultural consonance in social support	0.52 (± 0.16)	0.48 (± 0.13)	0.56 (± 0.18)
Cultural consonance in family life^∗^	101.8 (± 27.9)	95.3 (± 28.7)	107.9 (± 26.1)
Cultural consonance in national identity^∗∗∗^	44.9 (± 11.9)	48.7 (± 10.7)	41.3 (± 12.1)
Cultural consonance in occupation/education^∗∗∗^	68.0 (± 17.0)	61.9 (± 13.9)	73.8 (± 17.7)

*^a^SES, socioeconomic status. ^∗^*p* < 0.10, ^∗∗^*p* < 0.05, ^∗∗∗^*p* < 0.01.*

A correlation matrix of all variables is provided in Supplementary Table 2. There are a few noteworthy correlations. First, of the measures of cultural competence, there were only two with significant bivariate correlations with psychological distress: cultural competence in family life (*r* = -0.308, *p* < 0.05) and cultural competence in occupational and educational aspirations (*r* = -0.249, *p* < 0.05). With the exception of cultural consonance in social support, all measures of cultural consonance had significant bivariate correlations with psychological distress, ranging from relatively modest (*r* = -0.313, *p* < 0.05 for cultural consonance in lifestyle) to relatively large (*r* = -0.569, *p* < 0.01 for cultural consonance in occupational and educational aspirations).

The bivariate correlations of measures of cultural competence and measures of cultural consonance with psychological distress were compared using the method of Steiger (1980). The correlations with psychological distress of cultural consonance in family life and cultural competence in family life were significantly different (*p* < 0.10), as were the correlations of cultural consonance in national identity and cultural cynicism (*p* < 0.001), and the correlations of cultural consonance in occupational and education and cultural competence in occupational and education (*p* < 0.02).

It is also noteworthy that there was a significant correlation between cultural competence and cultural consonance in only one domain: social support (*r* = 0.638, *p* < 0.01).

Within-domain multiple regression analyses for each domain are shown in **Table 2**. The only associations of cultural competence with psychological distress are observed in the cultural domains of family life and occupational and educational aspirations, and these are relatively small associations (*p* < 0.10).

**Table 2:** Regression analysis within cultural domains (standardized regression coefficients, with unstandardized coefficients and standard errors shown in parentheses).

Cultural domain:	Lifestyle	Social support	Family life	Occ./Educ.	National identity
	Dependent variable – Psychological distress				
Age	0.077	0.022	0.087	0.223^∗^	–0.087
	(0.005 ± 0.01)	(0.002 ± 0.01)	(0.006 ± 0.82)	(0.016 ± 0.01)	(–0.006 ± 0.01)
Gender	–0.272^∗^	–0.255^∗∗^	–0.068	–0.160	–0.197^∗^
	(–0.611 ± 0.26)	(–0.574 ± 0.28)	(–0.153 ± 0.23)	(–0.359 ± 0.23)	(–0.442 ± 0.25)
Socioeconomic status	–0.304^∗^	–0.369^∗∗∗^	–0.233^∗∗^	–0.078	–0.230^∗^
	(–0.304 ± 0.12)	(–0.369 ± 0.12)	(–0.233 ± 0.10)	(–0.078 ± 0.12)	(–0.230 ± 0.12)
Cultural competence	–0.164	–0.237	–0.198^∗^	–0.207^∗^	–0.138
	(–0.696 ± 0.49)	(–1.31 ± 0.86)	(–1.69 ± 0.88)	(–0.433 ± 0.23)	(–0.597 ± 0.52)
Cultural consonance	–0.290^∗∗∗^	0.241	–0.481^∗∗∗^	–0.529^∗∗∗^	0.338^∗∗∗^
	(–0.014 ± 0.01)	(1.43 ± 1.1)	(–0.017 ± 0.01)	(–0.031 ± 0.01)	(0.028 ± 0.01)
Constant	1.41	0.033	2.83	1.50	–0.573
*R*	0.530^∗∗∗^	0.457^∗∗∗^	0.651^∗∗∗^	0.645^∗∗∗^	0.542^∗∗∗^
*R*^2^	0.281	0.209	0.423	0.416	0.293

*^∗^*p* < 0.10, ^∗∗^*p* < 0.05, ^∗∗∗^*p* < 0.01.*

With the exception of the domain of social support, higher cultural consonance in the cultural model is associated with psychological distress. As we have observed in other studies, higher cultural consonance is associated with lower psychological distress, with the exception of the domain of national identity in which that association is reversed. This is because cultural consonance in that domain assesses a higher cultural cynicism, which in turn is associated with greater distress.

### Analysis and Results Combining Cultural Domains

In an earlier study we found that multiple measures of cultural consonance load a single principal component (Dressler et al., 2007a). In a later study we confirmed that this is because they are culturally integrated around the construct of ‘goals in life’ (Dressler et al., 2017). In the current sample all five measures of cultural consonance load a single principal component that accounts for about 60% of the variance among measures (all measures have positive loadings on that component, with the exception of cultural cynicism, which has a negative loading). We refer to this principal component score as ‘cultural consonance in life goals.’

We also factor analyzed the five measures of cultural competence, which returned two factors (with varimax rotation), accounting for about 60% of the total variance among measures. Cultural competence in the domains of family life, social support, and national identity all loaded the first factor, which we will refer to as ‘social competence.’ Cultural competence in the domains of lifestyle and occupational and educational pursuits both loaded the second factor, which we will refer to as ‘status competence.’

A correlation matrix of all variables is presented in **Table 3**. Both social competence (*r* = -0.312, *p* < 0.01) and status competence (*r* = -0.228, *p* < 0.01) have modest inverse associations with psychological distress, while cultural competence in life goals (*r* = -0.668, *p* < 0.01) has a rather substantial inverse association with psychological distress. The other correlation worth noting here is the association of social competence and cultural consonance in life goals (*r* = 0.321, *p* < 0.01).

**Table 3:** Correlation matrix of psychological distress, covariates, cultural competence factors, and cultural consonance in life goals.

	Psychological distress	Age	Gender	SES	Social competence	Status competence	Cultural consonance
Psychological distress	–						
Age	0.017	–					
Gender	–0.202*	0.106	–				
SES	–0.360***	–0.121	–0.001	–			
Social competence	–0.312***	–0.278**	–0.069	0.308***	–		
Status competence	–0.228**	0.330**	–0.033	–0.043	0.000	–	
Cultural consonance in life goals	–0.668***	0.143	0.116	0.438***	0.321***	0.153	–

*^∗^*p* < 0.10, ^∗∗^*p* < 0.05, ^∗∗∗^*p* < 0.01.*

The bivariate correlations of cultural competence and cultural consonance with psychological distress were compared using the method of Steiger (1980). The correlation of cultural consonance in life goals with psychological distress was significantly larger than the correlation of social competence with psychological distress (*p* < 0.001). The correlation of cultural consonance in life goals with psychological distress was also significantly larger than the correlation of status competence with psychological distress (*p* < 0.002).

The regression of psychological distress on covariates, social competence, status competence, and cultural consonance in life goals is presented in **Table 4**. Status competence is associated with lower psychological distress (beta = -0.197, *p* < 0.05). This effect is, however, substantially smaller than the influence of cultural consonance in life goals (beta = -0.587, *p* < 0.01). A substantial portion of the variance in psychological distress is accounted for in this analysis (52%), and cultural consonance in life goals uniquely accounts for 24% of the variance.

**Table 4:** Regression analysis of psychological distress, covariates, cultural competence factors, and cultural consonance in life goals (standardized regression coefficients, with unstandardized coefficients and standard errors shown in parentheses).

Age	0.155 (0.011 ± 0.01)
Gender	–0.161^∗^ (-0.362 ± 0.21)
Socioeconomic status	–0.071 (–0.071 ± 0.11)
Social competence	–0.070 (–0.070 ± 0.11)
Status competence	–0.197^∗∗^ (-0.197 ± 0.10)
Cultural consonance in life goals	–0.587^∗∗∗^ (-0.587 ± 0.11)
Constant	–0.490
*R*	0.721^∗∗∗^
*R*^2^	0.520

*^∗^*p* < 0.10, ^∗∗^*p* < 0.05, ^∗∗∗^*p* < 0.01.*

Interaction effects between cultural consonance and the two measures of cultural competence were also assessed; neither of those interaction effects approached significance.

## Discussion

The aims of this paper, and the data on which it is based, are fairly modest. The aims were to examine the association of cultural competence, or individual knowledge of a cultural domain, with psychological distress, relative to the well-replicated association of cultural consonance and psychological distress. A second aim was to examine the associations of cultural competence and cultural consonance. The data to examine these associations comes from a modestly sized sample. At the same time, the analysis presented here rests on over 25 years of work on this topic in the setting of urban Brazil, and it takes advantage of that previous research, especially in terms of the measures employed. It therefore represents a small part of a much larger enterprise with respect to the study of culture, the individual, and psychological distress.

Generally speaking, with respect to psychological distress, it is good to be culturally competent in a cultural model, while it is better to be culturally consonant with that model. The results with respect to cultural competence are not particularly strong, and the results vary in relation to the cultural domain in which competence is measured. In the analyses on a domain-by-domain basis, once cultural consonance is controlled for, it is only in the domains of family life and occupational and educational aspirations that there are still (marginally) statistically significant associations of cultural competence with psychological distress. When the factor analysis of cultural competence measures is employed, the factor we have labeled ‘status’ competence, combining cultural competence in the domains of lifestyle and occupational and educational pursuits, has a small but statistically significant association with psychological distress while controlling for cultural consonance. This would suggest that Copeland’s (2017) findings with respect to the beneficial effect of cultural competence in the cultural model of HIV management in Kenya may be stronger if a measure of cultural consonance with the model was included.

There are several interesting aspects to the associations found in our study. Higher cultural competence, regardless of being able to act on that competence, may contribute to what Antonovsky (1979) referred to as a ‘sense of coherence.’ Antonovsky developed this notion when he pondered what it was that contributed to better health in human social life. A sense of coherence refers to an enduring but flexible worldview in which life is seen to be unfolding more-or-less as anticipated. The ‘more-or-less’ proviso is important, because Antonovsky never argued that everything in one’s life had to be going perfectly for one to have this worldview. But as long as one’s life seemed to be generally on track, this sense of coherence would develop, contributing to a sense of well-being. In previous work we have invoked Antonovsky’s ideas in relation to the salutary effects of higher cultural consonance, but they would seem to be apt for interpreting the association of cultural competence and distress as well. The individual who is competent in a cultural model at least has the sense that he or she understands the world around him or her, regardless of whether or not that understanding can be put into social practice. There is likely to be at least some benefit psychologically to understanding the way the social world works.

The pattern of the factor analysis of the cultural competence measures is interesting as well. Roberto da Matta, the Brazilian anthropologist who taught for many years in the United States, devoted much of his attention later in his career to Brazilian national character, with one of his more famous works entitled *A Casa e a Rua*, or ‘The Home and the Street’ (Da Matta, 1985). His main aim in that work was to describe gendered social relations in Brazil. The metaphor of home and street describes the domains of women and men, respectively. The home is a place of safety, security, and even a kind of purity that is the domain of women. The street carries risk and impurity and is the domain in which men compete for status and success. What we have labeled social competence combines measures of cultural competence from the domains of family life, social support, and Brazilian identity. Recall that the latter measure, which we dubbed cultural cynicism, deals exclusively with how Brazilians treat one another in mundane interaction. These domains thus all describe personal social interaction, with the family dominating the factor, and refer to da Matta’s domain of *a casa*. The second factor, status competence, combines cultural competence in lifestyle and occupational and educational pursuits, both domains that project one’s place in the social status hierarchy in mundane social interaction and hence refers to da Matta’s domain of *a rua*. Both social and status competence have moderately strong bivariate correlations with psychological distress; however, only status competence retains a significant association with psychological distress once cultural consonance in life goals is controlled for. Again, simply having greater understanding of the cultural consensus model in these domains is associated with lower psychological distress.

With one exception (the cultural domain of social support), there are no associations between cultural competence and cultural consonance in the same domain. This is actually not surprising, in part because of the statistical properties of cultural competence. Recall that cultural consensus analysis is designed to identify the shared model in a given domain. If there is a shared model, then each individual’s cultural competence in that model will be fairly high, and the distribution of competence coefficients will have a right-hand skew. As Hruschka and Maupin (2013) have shown in a simulation study, the differences among respondents in cultural competence in such a situation can be little more than ‘noise’ in the data, since there is substantial agreement. In the data presented here, all of the distributions of cultural competence are skewed to the right, which is apparent on visual inspection, although actual values of skewness do not exceed -1.0 in any domain, which justifies their continued use in multiple regression analysis. What this means is that there is comparatively less variation in knowledge of a domain relative to the substantial variation in actually achieving high cultural consonance in that domain. Put differently, most people know fairly well what is prototypical (and hence, in a sense, ‘ideal,’ see Burnett et al., 2005) in a given cultural domain. What varies substantially is their ability to put that knowledge into practice, which is more strongly influenced by other factors, such as socioeconomic status (the bivariate correlations of cultural consonance and household income in different domains range from a low of *r* = 0.26 in the domain of family life to a high of *r* = 0.58 in the domain of lifestyle, see Dressler et al., 2017). Therefore, individual knowledge in a given domain is simply not that strong a predictor of behavior, since what is important is collective knowledge, not individual representations (see Dressler, 2018).

As noted, the one exception to the lack of correlation of competence and consonance is in the domain of social support. This probably confirms what we have suspected for some time, and that is that our measure of cultural consonance in social support actually assesses something closer to cultural competence. The respondents in each task are asked to rank order potential sources of social support in response to hypothetical problems. The only difference in the two tasks is that in the data collected for CCA they are instructed to think in terms of the community at large (i.e., how people ‘around here’ would rank them), and in the data collected for the cultural consonance measure they are instructed to ask in terms of their own personal actions. It is not unreasonable to conclude here that, when they are asked to imagine their behavior in these hypothetical situations, they respond in terms of their knowledge of the cultural model, as opposed to their own individual preferences. If this is true, a better way of measuring cultural consonance in social support needs to be developed.

With respect to the lack of correlation between cultural competence and cultural consonance, some readers may be tempted to view this as a process of cognitive dissonance (Festinger, 1957). That is, an individual unable to achieve higher cultural consonance in a domain would employ a dissonance reduction strategy and would come to reject the cultural consensus model in favor of an alternative—and dissonance reducing—personal model. This is a thorny issue that Dressler (2018) examines in some detail. Suffice it to say that this is probably easier said than done, given that the focus here is on broadly shared cultural models and consonance with them. To accomplish a dissonance reduction strategy would require literally stepping outside the normative space of social and cultural expectation, which would demand a critical reflexivity difficult for many. This is not to say it is impossible, and Snodgrass et al. (2014) offer some evidence for a cultural dissonance and its reduction in the cultural domain of online gaming. More research on this topic is thus needed.

Finally, the research reported here contributes an additional replication of the cultural consonance hypothesis. The association of cultural consonance in life goals and psychological distress, controlling for covariates and for cultural competence, is substantial, and the magnitude of the association is comparable to that observed in other samples (Dressler et al., 2007a, 2017). As the data presented here show, the magnitude of the effect of cultural consonance in life goals on psychological distress is in large part a function of the fact that cultural consonance in these different domains forms a latent variable. It is thus clearly important to understand how an individual’s cultural consonance extends across these multiple domains. In this case, the convergence of consonance in multiple domains is linear and additive. This is not, however, the case in all domains, as evidenced by the work of Snodgrass et al. (2013) and Dengah (2014). Dengah examined cultural consonance with religious ideals among Pentecostal Protestants in Brazil. One critical element of conversion from Catholicism to one of several variants of Pentecostalism is social class; originally (although this is changing), converts were drawn from the lower class as they sought a better life, materially, for themselves. It would be plausible to suppose that religious consonance would thus compensate for lack of cultural consonance in other domains, but this proved not to be the case. Rather, the effects of religious and secular consonance (specifically in the cultural domains of lifestyle and family life) were synergistic. The higher one’s religious consonance, the stronger the effect of secular consonance on psychological distress.

Snodgrass et al. (2013) on the other hand, examined ideas of success in the virtual world of online gaming and in the ‘real’ world of everyday life. They found that higher cultural consonance with success in the real world was associated with a lower likelihood of internet addiction, while cultural consonance with success in the virtual world was associated with a higher likelihood of internet addiction. Curiously, the two measures of cultural consonance were uncorrelated. In this empirical example, cultural consonance in two similar domains (i.e., ‘success,’ but in different contexts) is not only unrelated, it also has different implications for mental health.

We bring up this point and these studies because of a clear direction in research on cultural consonance that they suggest. Our research, and that of Dengah and Snodgrass, show that cultural models vary in the extent of their social distribution. Furthermore, cultural consonance in those domains may combine as a latent variable (our research), or cultural consonance in different domains may interact (as in Dengah’s, 2014) research, or cultural consonance in similar domains in different context may have opposite effects (as in Snodgrass et al., 2013). We all inhabit multiple social worlds and hence all face the potential for the cultural models associated with those worlds to reinforce one another, or to come into conflict. A refined understanding of culture and the individual will require more research in this area.

The major limitation of this study is its relatively modest sample size, drawn as it is from a previous sample, which itself would suffer from some kind of unknown sampling bias. Nevertheless, the sample employed here does include the range of socioeconomic variation in Brazilian society and therefore the estimates of the effect of cultural consonance are probably accurate. In the future, similar research should be carried out on larger and more representative samples.

We as individuals live in a social milieu that is structured by systems of shared meaning and understanding referred to as culture. A cognitive theory of culture provides an effective set of conceptual and methodological tools for examining that meaning and understanding at both the collective and individual levels. Continued work in this area may shed new insights in the study of culture and the individual.

## Ethics Statement

This study was carried out in accordance with the recommendations of the Institutional Review Board for the protection of human subjects of The University of Alabama with written informed consent from all subjects. All subjects gave written informed consent in accordance with the Declaration of Helsinki. The protocol was approved by the Institutional Review Board of The University of Alabama and the Ethics Committee, Faculty of Medicine, University of Sao Paulo-Ribeirao Preto.

## Author Contributions

WD designed the research and was responsible for data analysis and the writing of the manuscript. MB directed the fieldwork and contributed to the writing of the manuscript. JdS was administrator of the research in Brazil and contributed to the writing of the manuscript.

## Conflict of Interest Statement

The authors declare that the research was conducted in the absence of any commercial or financial relationships that could be construed as a potential conflict of interest.
