# Supplementary material for: What You Know, What You Do, and How You Feel: Cultural Competence, Cultural Consonance, and Psychological Distress
Source: Front Psychol. 2018 Jan 15;8:2355. doi: 10.3389/fpsyg.2017.02355 (PMC5775295; doi:10.3389/fpsyg.2017.02355)
Supplement: Supplementary file 1 [file Table_1.DOCX]

Supplementary Table 1: Specifics of the cultural domain analyses

|  | Cultural domain: | | | | |
| --- | --- | --- | --- | --- | --- |
| Research step: | Lifestyle | Social support | Family life | National Identitity | Occupational and Educational Aspirations^+^ |
| Free lists - 2001  (to generate elements in each domain) | n (subjects) = 43  n (items) = 146 | n (subjects) = 43  n (items) = 90 | n (subjects) = 43  n (items) = 145 | n (subjects) = 43  n (items) = 133 | n (subjects) = 41  n (items) = 22 |
| Pile sorts - 2001  (to discover salient distinctive features within each domain) | n (subjects = 40)  n (items) = 42 | n (subjects = 40)  n (items) = 14 | n (subjects) = 40  n (items) = 23 | n (subjects) = 40  n (items) = 26 | n (subjects) = 36  n (items) = 13 |
| Rating Tasks - 2001  (to test for cultural consensus in each domain) | n (subjects) = 66  n (items) = 32  eigenvalue  ratio = 6.59  Comp* = .71 (± .12) | n (subjects) = 66  n (items) = 16  eigenvalue  ratio =6.53  Comp* = .67 (± .14) | n (subjects) = 66  n (items) = 13  eigenvalue  ratio = 7.42  Comp* = .82 (± .09) | n (subjects) = 66  n (items) = 19  eigenvalue  ratio = 3.97  Comp* = .57 (± .19) | n (subjects) = 36  n (items) = 13  eigenvalue  ratio =  Comp* = .50 (± .17) |
| Replication of Cultural Consensus - 2011 | n (subjects) = 40  eigenvalue  ratio = 7.70  Comp* = .72 (± .11) | n (subjects) = 40  eigenvalue  ratio =5.21  Comp* = .65 (± .16) | n (subjects) = 40  eigenvalue  ratio = 9.62  Comp* = .84 (± .09) | n (subjects) = 40  eigenvalue  ratio = 3.50  Comp* = .61 (± .16) | --- |

^+^Note that the cultural domain analysis for occupational and educational aspirations was only carried out in 2011. For simplicity this information has been included along with the other cultural domain analyses

*Comp = Mean cultural competence (± s.d.)
